# Clinical valve thrombosis and arterial embolism in a cancer patient after transcatheter aortic valve replacement

**DOI:** 10.1093/omcr/omad125

**Published:** 2023-11-28

**Authors:** Shinnosuke Sawano, Mizuki Miura, Yasutomi Higashikuni, Hiroyuki Saigusa, Satoshi Kodera, Norifumi Takeda, Masaru Hatano, Jiro Ando, Minoru Ono, Issei Komuro

**Affiliations:** Department of Cardiovascular Medicine, Graduate School of Medicine, The University of Tokyo, Tokyo, Japan; Department of Cardiovascular Medicine, Graduate School of Medicine, The University of Tokyo, Tokyo, Japan; Department of Cardiovascular Medicine, Graduate School of Medicine, The University of Tokyo, Tokyo, Japan; Department of Radiology, The University of Tokyo Hospital, Tokyo, Japan; Department of Cardiovascular Medicine, Graduate School of Medicine, The University of Tokyo, Tokyo, Japan; Department of Cardiovascular Medicine, Graduate School of Medicine, The University of Tokyo, Tokyo, Japan; Department of Cardiovascular Medicine, Graduate School of Medicine, The University of Tokyo, Tokyo, Japan; Department of Cardiovascular Medicine, Graduate School of Medicine, The University of Tokyo, Tokyo, Japan; Department of Cardiac Surgery, Graduate School of Medicine, The University of Tokyo, Tokyo, Japan; Department of Cardiovascular Medicine, Graduate School of Medicine, The University of Tokyo, Tokyo, Japan

**Keywords:** anticoagulation, aortic valve, atrial fibrillation, cancer, thrombosis, valve replacement

## Abstract

The number of cancer patients with severe aortic stenosis and atrial fibrillation (AF) is increasing in the aging population. Transcatheter aortic valve replacement (TAVR) is an established treatment option for severe aortic stenosis with high surgical risk, including individuals with cancer. Antithrombotic therapy should be considered for post-TAVR or AF patients. However, antithrombotic management in cancer patients remains challenging due to the increased risk of both thromboembolism and bleeding. We present a case of clinical valve thrombosis and arterial embolism after transcatheter aortic valve replacement in an elderly patient with a history of metastatic pancreatic cancer and permanent atrial fibrillation under treatment of single antiplatelet therapy. Warfarin treatment after successful surgical thrombectomy to the occluded arteries improved clinical valve thrombosis, although the long-term outcome remains unclear. This case demonstrates that novel management algorithms for thromboembolism and bleeding in elderly cancer patients with AF and valvular heart disease are urgently needed.

## INTRODUCTION

The number of cancer patients with severe aortic stenosis (AS) and atrial fibrillation (AF) is increasing in the aging population. Transcatheter aortic valve replacement (TAVR) is an established treatment option for severe AS that enables mechanical intervention with a less invasive procedure than surgical aortic valve replacement in patients at high surgical risk, including cancer patients [1]. To prevent transcatheter aortic valve (THV) thrombosis, the current guidelines recommend lifelong single antiplatelet therapy (SAPT) for patients after TAVR; oral anticoagulation therapy is recommended for those who have other indications for anticoagulation [1]. In elderly patients with AF, anticoagulant therapy is indicated for prevention of left atrium (LA) thrombus formation [2]. Advanced cancer is associated with an increased risk of both thromboembolism and bleeding [3]. It remains largely unknown how thromboembolic and bleeding risk should be managed in post-TAVR and/or AF patients with advanced cancer.

## CASE REPORT

An 81-year-old man with a history of metastatic pancreatic cancer, permanent AF and TAVR due to symptomatic severe AS was referred to our hospital because of pain in the right upper and lower limbs during exercise. The patient had undergone TAVR with a 26-mm SAPIEN 3 valve (Edwards Lifesciences, Irvine, California, USA) 2 years earlier. After TAVR, the patient had received rivaroxaban for the first 3 months, and then rivaroxaban was switched to clopidogrel, considering higher bleeding risk in patients with metastatic pancreatic cancer than in those without [4, 5].

**Figure 1:**
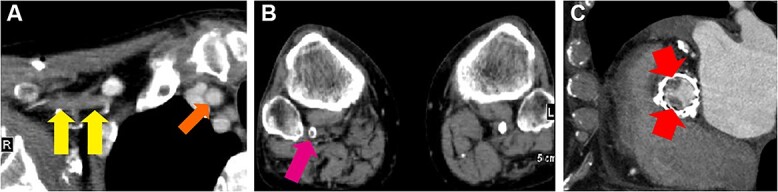
Contrast-enhanced computed tomographic images. Reduced contrast enhancement of the occluded right subclavian artery (**A**, two arrows on the left side) compared with that of the right common carotid artery (A, arrow on the right side), filling defect of the right popliteal artery (**B**, arrow) and transcatheter heart valve thrombosis (**C**, arrows) were detected.

**Figure 2:**
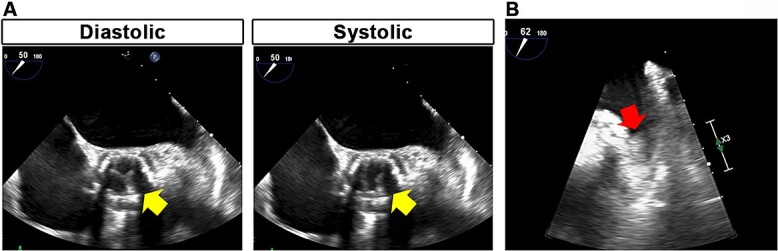
Transesophageal echocardiographic images. Reduced leaflet motion of the bioprosthetic valve (**A**, arrows) and left atrial appendage thrombus (**B**, arrow) were detected.

On physical examination, the right upper and lower extremities were pale with weak pulses. Extremity blood pressures were 147/80 mmHg in the left arm, 78/- mmHg in the right arm, 166/70 mmHg in the left ankle, and not detectable in the right ankle. Laboratory data showed elevated levels of white blood cell count (9.5 × 10^4^/μl; reference range, 3.3–8.6 × 10^4^/μl), lactate dehydrogenase (311 U/ml; reference range, 124–222 U/ml), D-dimers (5.2 μg/ml; reference range, <1.0 μg/ml) and CA19-9 (93 U/ml; reference range, <36 U/ml), while the serum levels of creatine kinase, potassium, creatinine and carcinoembryonic antigen were within the reference range. Contrast-enhanced computed tomography (CT) was performed, revealing reduced contrast enhancement of the right subclavian artery compared with that of the right common carotid artery, indicating an occlusion of the right subclavian artery (Fig. 1A). In addition, a filling defect was detected in the right popliteal artery (Fig. 1B). Furthermore, thrombi were found in two out of three bioprosthetic leaflets with a diameter of approximately 10 mm (Fig. 1C). Transthoracic and transesophageal echocardiography showed reduced leaflet motion of the bioprosthetic valve (Fig. 2A) and an increased mean pressure gradient (MPG) across the aortic valve (AV) of 26.3 mmHg with a peak velocity of 3.4 m/s, compared with those at baseline after TAVR with an AV MPG of 8.0 mmHg and a peak velocity of 2.14 m/s, which suggested clinical valve thrombosis. In addition, a thrombus was observed in the LA appendage (Fig. 2B). Collectively, the patient was diagnosed with arterial embolism in the right upper and lower extremities and clinical valve thrombosis.

**Figure 3:**
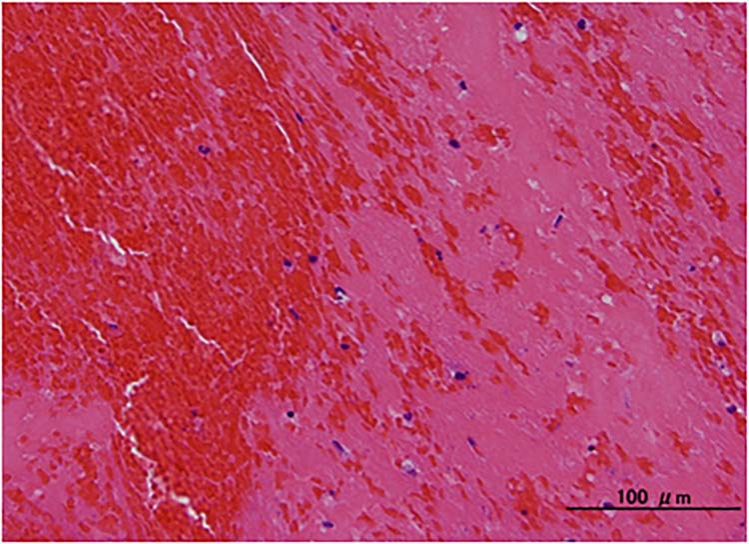
Histological finding. Hematoxylin and eosin staining revealed that surgically removed thrombi were rich in fibrin and red blood cells.

The patient electively underwent successful Fogarty balloon catheter embolectomy to the right subclavian artery and percutaneous transluminal angioplasty to the right popliteal artery. Histological assessment revealed that surgically removed thrombi were rich in fibrin and red blood cells (Fig. 3), which provided supportive evidence for embolic events in this case. The patient received warfarin monotherapy with a target international normalized ratio range of 2.0–3.0. After 5 months of follow-up, transthoracic echocardiography showed that an AV MPG was decreased to 6.6 mmHg with a peak velocity of 1.7 m/s.

## DISCUSSION

Accumulating evidence has shown that the risk of bleeding and thromboembolism varies across different cancer types [5]. Pancreatic cancer is associated with higher risk of both cancer-associated thrombosis and major bleeding with high morbidity and mortality, compared with other types of cancer [4–6]. How cancer type-specific bleeding and thromboembolic risk should be incorporated into clinical practice remains undefined.

Symptomatic THV thrombosis, also called clinical valve thrombosis, is a rare complication after TAVR, with an incidence between 0.6% and 2.8% [7–9]. The risk of stroke was higher in patients with clinical valve thrombosis than in those without [7]. In the present case, symptomatic THV thrombosis occurred while on treatment with SAPT. SAPT might not be sufficient for the prevention of THV thrombosis in a certain population of cancer patients, although AF might contribute to THV thrombosis in this case. Warfarin was effective for the treatment of clinical valve thrombosis in this patient, as recommended by the guidelines [1]. In this case, warfarin was continued because the effectiveness of direct oral anticoagulants on prevention of THV and LA thrombosis in this patient was unclear. However, the optimal antithrombotic therapy for the long-term follow-up in post-TAVR patients with cancer remains unclear.

Several large clinical trials have shown that there was no significant difference in the incidence of stroke or systemic embolic events between AF patients with and without active cancer, whereas the bleeding risk in elderly AF patients with active cancer was reported to be higher than in those without [10]. In this case, currently available risk scoring systems in the general AF population, such as the CHA2DS2VASc, HAS-BLED and ORBIT scores, indicated that the patient had stroke risk warranting anticoagulation and low bleeding risk, with 3, 2 and 2 points, respectively. However, these scores have not been sufficiently validated in patients with cancer [5]. Indeed, the HAS-BLED score was reported to underestimate the bleeding risk in patients with AF and concomitant cancer [4]. Considering the higher bleeding risk in cancer patients, careful consideration of the indication of anticoagulation therapy was given in this case.

This case demonstrates that novel algorithms, which would predict the risk of thromboembolic and bleeding events based on cancer type, stage, activity and treatment, are urgently needed to individually optimize antithrombotic therapy in post-TAVR AF patients with cancer. In addition, further studies are necessary to examine whether post-TAVR AF patients with suboptimal antithrombotic therapy due to cancer-related bleeding risk benefit from frequent follow-up, including routine contrast-enhanced CT, for prevention of serious thromboembolic events.

## FUNDING

None.

## ETHICAL APPROVAL

Not applicable.

## CONSENT

Written informed consent was obtained from the patient for the publication of this case report and accompanying images.

## GUARANTOR

Dr. Yasutomi Higashikuni.

## CONFLICT OF INTEREST STATEMENT

None declared.

## DATA AVAILABILITY

The data underlying this article are available in the article.

## References

[1] Otto CM, Nishimura RA, Bonow RO, Carabello BA, Erwin JP 3rd, Gentle F. et al. 2020 ACC/AHA guideline for the Management of Patients with valvular heart disease: a report of the American College of Cardiology/American Heart Association joint committee on clinical practice guidelines. Circulation 2021;143:e72–227. 33332150 10.1161/CIR.0000000000000923

[2] January CT, Wann LS, Calkins H, Chen LY, Cigarroa JE, Cleveland JC Jr. et al. 2019 AHA/ACC/HRS focused update of the 2014 AHA/ACC/HRS guideline for the management of patients with atrial fibrillation: a report of the American College of Cardiology/American Heart Association task force on clinical practice guidelines and the Heart Rhythm Society in collaboration with the Society of Thoracic Surgeons. Circulation 2019;140:e125–51. 30686041 10.1161/CIR.0000000000000665

[3] Lyman GH, Carrier M, Ay C, Di Nisio M, Hicks LK, Khorana AA. et al. American Society of Hematology 2021 guidelines for management of venous thromboembolism: prevention and treatment in patients with cancer. Blood Adv 2021;5:927–74. 33570602 10.1182/bloodadvances.2020003442 PMC7903232

[4] Pastori D, Marang A, Bisson A, Menichelli D, Herbert J, Lip GYH. et al. Thromboembolism, mortality, and bleeding in 2,435,541 atrial fibrillation patients with and without cancer: a nationwide cohort study. Cancer 2021;127:2122–9. 33631041 10.1002/cncr.33470

[5] Pastori D, Marang A, Bisson A, Herbert J, Lip GYH, Fauchier L. Performance of the HAS-BLED, ORBIT, and ATRIA bleeding risk scores on a cohort of 399 344 hospitalized patients with atrial fibrillation and cancer: data from the French National Hospital Discharge Database. J Am Heart Assoc 2022;11:e026388. 36444864 10.1161/JAHA.121.026388 PMC9851457

[6] Khorana AA, Fine RL. Pancreatic cancer and thromboembolic disease. Lancet Oncol 2004;5:655–63. 15522652 10.1016/S1470-2045(04)01606-7

[7] Rheude T, Pellegrini C, Stortecky S, Marwan M, Xhepa E, Ammon F. et al. Meta-analysis of bioprosthetic valve thrombosis after transcatheter aortic valve implantation. Am J Cardiol 2021;138:92–9. 33065085 10.1016/j.amjcard.2020.10.018

[8] Latib A, Naganuma T, Abdel-Wahab M, Danenberg H, Cota L, Barbanti M. et al. Treatment and clinical outcomes of transcatheter heart valve thrombosis. Circ Cardiovasc Interv 2015;8:e001779. 25873727 10.1161/CIRCINTERVENTIONS.114.001779

[9] Jose J, Sulimov DS, El-Mawardy M, Sato T, Allali A, Holy EW. et al. Clinical bioprosthetic heart valve thrombosis after transcatheter aortic valve replacement: incidence, characteristics, and treatment outcomes. JACC Cardiovasc Interv 2017;10:686–97. 28385406 10.1016/j.jcin.2017.01.045

[10] Yasui T, Fujita M. Anticoagulation for atrial fibrillation in patients with cancer. Circ J 2022;86:211–2. 34980784 10.1253/circj.CJ-21-1003

